# SARS-CoV-2 epidemiological trend before vaccination era: a seroprevalence study in Apulia, Southern Italy, in 2020

**DOI:** 10.1007/s10389-023-01834-3

**Published:** 2023-02-04

**Authors:** Serena Marchi, Chiara Coppola, Pietro Piu, Linda Benincasa, Francesca Dapporto, Alessandro Manenti, Simonetta Viviani, Emanuele Montomoli, Claudia Maria Trombetta

**Affiliations:** 1 Department of Molecular and Developmental Medicine, University of Siena, 53100 Siena, Italy; 2 VisMederi srl, 53100 Siena, Italy; 3 VisMederi Research srl, 53100 Siena, Italy

**Keywords:** SARS-CoV-2, seroprevalence, Italy, Apulia region

## Abstract

**Aim:**

The present study aimed at assessing the prevalence of antibodies against SARS-CoV-2 in the general population in the province of Bari (Apulia region, Southern Italy) during the year 2020.

**Subject and methods:**

In this study, 1325 serum samples collected from January to December 2020 were tested for the presence of IgM and IgG antibodies against whole-virus SARS-CoV-2 antigen by commercial ELISA. Positive samples were further tested by in-house ELISA for the detection of anti-receptor binding domain (RBD) IgM and IgG antibodies and by micro-neutralization (MN) assay for the detection of neutralizing antibody.

**Results:**

One hundred (7.55%) samples had the presence of at least one antibody class against SARS-CoV-2 by commercial ELISA, of which 88 (6.6%) showed IgG and 19 (1.4%) showed IgM antibodies. The proportion of samples with IgG antibodies increased from 1.9% in January–February to 9.6% in November–December, while no significant increase was observed for IgM. When tested by in-house ELISA and MN assay, 17.0% and 31.6% were found positive to RBD IgG and RBD IgM, respectively, while 12.0% showed neutralizing antibody.

**Conclusion:**

The proportion of samples with SARS-CoV-2 IgG antibodies increased during 2020, especially in the second half of the year, consistent with data reported by the routine epidemiological surveillance of SARS-CoV-2 cases. Despite the high number of reported cases, the seroprevalence values are relatively low, and only a small proportion of samples had neutralizing antibodies.

**Supplementary Information:**

The online version contains supplementary material available at 10.1007/s10389-023-01834-3.

## Introduction

After the first cases of SARS-CoV-2 infection were recorded in the northern part of Italy, on 9 March 2020 a national lockdown (Phase 1) was declared, lasting until 3 May 2020 (Presidenza del Consiglio dei Ministri - Governo Italiano 2020). The first pandemic wave, which lasted from the end of February to the beginning of May, presented an extremely heterogeneous geographical picture with major involvement of the Northern regions (Dorrucci et al. 2021) of Italy. Following the decrease in infections, containment measures were gradually relaxed from 4 May (Phase 2) and on 15 June with the restoration of national mobility (Phase 3) (Presidenza del Consiglio dei Ministri - Governo Italiano 2020). From the end of September, a second pandemic wave occurred, involving Italy more evenly from north to south. With the decree of 6 November 2020, Italian regions were divided into three risk areas (yellow, orange, and red) characterized by increasing levels of containment according to COVID-19 mortality and morbidity (Presidenza del Consiglio dei Ministri - Governo Italiano 2020).

**Fig. 1:**
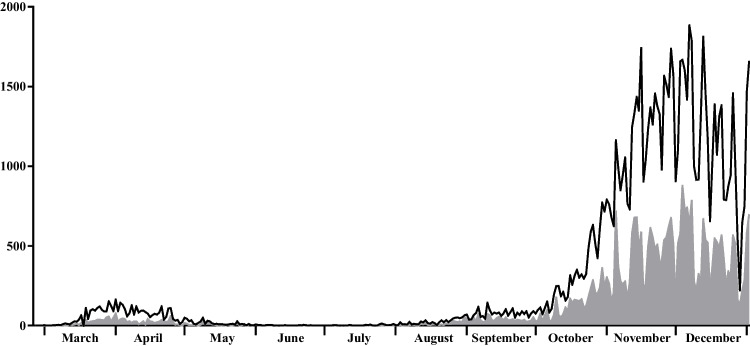
New SARS-CoV-2 infection cases from 27 February to 31 December 2020 in the Apulia region (continuous black line) and in the province of Bari (grey filled line), according to the Italian Department of Civil Protection (Presidenza del Consiglio dei Ministri 2020)

The first case of infection in Apulia was reported on 27 February 2020. During the first pandemic wave, the highest number of cases recorded in Apulia in 24 hours was reached on 30 March 2020 with 163 cases (Presidenza del Consiglio dei Ministri 2020) (Fig. 1), followed by a progressive decrease during spring and summer. From September, a rapid increase in cases was observed (Istituto Superiore di Sanità 2021a), thus marking the initiation of the second pandemic wave in Apulia. The impact of the second wave in terms of daily cases was substantially higher than during the first wave, with 1884 cases recorded in 24 hours on 5 December (Presidenza del Consiglio dei Ministri 2020) (Fig. 1). According to the decree of 6 November, the Apulia region was classified as an “orange zone” until 6 December and “yellow zone” until 21 December, when further social containment measures were implemented throughout the national territory until the beginning of 2021 (Presidenza del Consiglio dei Ministri - Governo Italiano 2020).

In Italy, the COVID-19 vaccine distribution started on 31 December 2020. The vaccine was offered firstly to all healthcare workers on the front line of the COVID-19 response, and all people working on-site at public or private social and healthcare facilities, followed by individuals older than 80 years (Istituto Superiore di Sanità 2021b). The present study aimed at assessing the prevalence of antibodies against SARS-CoV-2 in a sample population in the province of Bari, Apulia region, Italy, during 2020 in order to estimate the seroprevalence of SARS-CoV-2 in a population before the vaccination era when only social containment measures had been implemented.

## Methods

### Study population

Human serum samples were anonymously collected from January to the end of December 2020 from a local laboratory in a small town of about 60,000 inhabitants in the province of Bari, Apulia region, Southern Italy, as residual samples for unknown diagnostic purposes and stored at the laboratory of Molecular Epidemiology of the University of Siena, Italy, in compliance with Italian ethics law (Presidenza del Consiglio dei Ministri 2009). For each sample, only information on age, sex, pregnancy status, and month of collection was available.

Sample size was calculated assuming a precision of the estimate of 2% with a confidence interval of 95% (95% CI) and an overall SARS-CoV-2 antibody prevalence of 0.99% (Fiore et al. 2021).

A total of 1325 serum samples were randomly selected by bimester, from January–February to November–December. Further, samples were stratified by sex and age group (≤19, 20–29, 30–39, 40–49, 50–59, 60–69, 70–79, ≥80 years). Women of childbearing age (15–49 years) (World Health Organization 2022) were also stratified according to pregnancy status.

### Serological assays

Samples were tested by commercial enzyme-linked immunosorbent assay (ELISA; Enzywell SARS-CoV-2 IgM and IgG, DIESSE, Siena, Italy) for the detection of immunoglobulin (Ig) M and IgG antibodies against SARS-CoV-2, using ELISA plates coated with inactivated whole-virus SARS-CoV-2 antigen. According to the manufacturer’s instructions, samples were considered positive when the ratio between the optical density (OD) of the sample and that of the cut-off was >1.1, negative if the ratio was <0.9, and borderline if the ratio was between 0.9 and 1.1.

Samples with a borderline or positive result for IgG and/or IgM were further tested by an in-house ELISA for the detection of IgG and IgM against the receptor-binding domain (RBD) of the spike (S) protein and by micro-neutralization (MN) assay for the detection of neutralizing antibody.

The in-house ELISA was performed as previously reported (Mazzini et al. 2021). Briefly, ELISA plates (Nunc, Maxi-Sorp) were coated with 1 μg/mL of purified recombinant spike-RBD HEK-derived protein (Sino Biological, China). Human serum samples were diluted 1:100 in TBS-0.05% Tween 20-5%, and then 100 μL of each serum dilution was added to the coated plates and incubated for 1 hour at 37 °C. After the washing step, goat anti-human IgG-Fc or IgM μ-chain horseradish peroxidase (HRP)-conjugated antibody (Bethyl Laboratories, Montgomery, TX, USA) was added and plates incubated at 37 °C for 30 minutes. After the washing step, 3,3′,5,5′-tetramethylbenzidine (TMB) substrate (Bethyl Laboratories, Montgomery, TX, USA) was added and incubated in the dark at room temperature for 20 minutes. The reaction was stopped and read at 450 nm. A cut-off value was defined as three times the average optical density (OD) values from blank wells (background: no addition of analyte). Samples with OD below the cut-off value were classified as negative, while samples with OD above the cut-off value were classified as positive (Milani et al. 2020).

The MN assay was performed as previously reported (Manenti et al. 2020) and using wild-type SARS-CoV-2 (2019-nCov/Italy-INMI1 strain) virus, purchased from the European Virus Archive—Global (EVAg, Spallanzani Institute, Rome). Briefly, serum samples were heat-inactivated for 30 minutes at 56 °C and twofold serially diluted (starting dilution 1:10), then mixed with an equal volume of SARS-CoV-2 viral solution containing 100 tissue culture infective dose 50% (TCID_50_). After 1 hour of incubation at room temperature, 100 μL of each virus-serum mixture was added to a 96-well plate containing an 80%-confluent Vero E6 cell monolayer. Plates were incubated for 72 hours at 37 °C, 5% CO_2_ in humidified atmosphere, then inspected for presence/absence of cytopathic effect (CPE) by means of an inverted optical microscope. The highest sample dilution showing no signs of CPE was regarded as the neutralization titre.

### Statistical analysis

Crude and adjusted prevalence of SARS-CoV-2 IgG and IgM antibodies by each month during the study period were calculated (Lang and Reiczigel 2014). For the calculation of the seroprevalence adjusted for the specificity and sensitivity of the commercial ELISAs, 92.5% sensitivity and 95.8% specificity for IgG ELISA and 87.7% sensitivity and 97.0% specificity for IgM ELISA were considered, as reported by the manufacturer. The adjusted prevalence was calculated using the following formula:

$${P}_{adj}=\frac{P-\left(1- SPE\right)}{SEN+ SPE-1}$$

The adjusted prevalence could theoretically assume negative values if the crude prevalence was less than the expected false positive rate. To address this issue, negative adjusted values were reported as zero.

A test for equality of proportions was used to evaluate differences in adjusted prevalence by bimester and age group. The 95% CIs of the adjusted prevalence by bimester were calculated using the Agresti–Coull method. Statistical significance was set at *p*<0.05.

## Results

The median age of the study population was 50.0 years, with a range of 3–99 years; 849 (64.1%) samples were from female individuals and 476 (35.9%) from males. Out of 466 samples collected from women of childbearing age, 184 (39.5%) were from pregnant women.

**Fig. 2:**
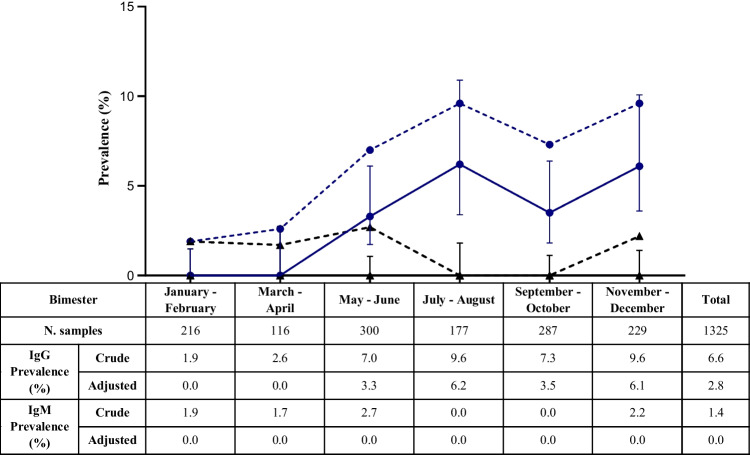
Number of samples tested by commercial ELISA and prevalence by antibody class. Dashed lines indicate IgG (blue circle) and IgM (black triangle) crude prevalence, continuous lines indicate IgG (blue circle) and IgM (black triangle) adjusted prevalence, with 95% confidence intervals

Of the 1325 samples included in the study and tested by commercial ELISA, a total of 100 (7.55%) had the presence of at least one antibody class against SARS-CoV-2, of which 88 (6.6%) showed IgG and 19 (1.4%) showed IgM antibodies. Among these positive samples, 7 (0.5%) samples showed both IgG and IgM antibodies (Fig. S1). The proportion of samples with SARS-CoV-2 IgG antibodies increased during the study period (Fig. 2). In January–February, 1.9% (adjusted prevalence, 0.0%) were positive to IgG, and 9.6% (adjusted prevalence, 6.1%) were positive in November-December (*p*=0.0008). No significant increase was observed for IgM, whose prevalence was 1.9% (adjusted prevalence, 0.0%) in January–February and 2.2% (adjusted prevalence, 0.0%) in November–December (Fig. 2).

IgG antibodies were found in 57 women (6.7%, adjusted prevalence 2.8%) and 31 males (6.5%, adjusted prevalence 2.6%), while IgM antibodies were found in 14 women (1.6%, adjusted prevalence 0.0%) and 5 males (1.0%, adjusted prevalence 0.0%). Among pregnant women, IgG and IgM prevalence were 6.5% (adjusted prevalence, 2.6%) and 0.5% (adjusted prevalence, 0.0%), respectively, similar to those observed in women of the same age (IgG prevalence 6.4%, adjusted prevalence 2.5%; IgM prevalence 2.5%, adjusted prevalence 0.05). IgG and IgM prevalence by age group is shown in Table 1. No differences were found by sex, pregnancy status, or age group.

**Table 1:** Crude and adjusted prevalence of commercial ELISA IgG and IgM, by age group

Age group (years)		≤19	20–29	30–39	40–49	50–59	60–69	70–79	≥80
No. samples		79	177	248	145	160	174	206	136
IgG prevalence (%)	Crude	6.3	7.3	7.3	6.9	6.2	4.0	7.8	6.6
	Adjusted	2.4	3.6	3.5	3.0	2.3	0.0	4.0	2.7
IgM prevalence (%)	Crude	0.0	3.4	0.4	1.4	0.6	1.1	3.4	0.0
	Adjusted	0.0	0.5	0.0	0.0	0.0	0.0	0.5	0.0

IgG and IgM positive or borderline samples by commercial ELISA were further tested by RBD-based ELISA and MN assay. Fifteen (15) out of 88 (17.0%) and 6 out of 19 (31.6%) samples were found positive to RBD IgG and RBD IgM, respectively. Of these positive samples, 3 samples were positive to both RBD IgG and RBD IgM. When tested by MN assay, 12 out of 100 (12.0%) samples showed neutralizing antibody (antibody titer range 10–1280) (Fig. S1). In particular, 11 out of 18 (61.1%) samples positive to RBD IgG and/or IgM showed neutralizing antibodies, while all but one sample negative to RBD antibodies were also negative to MN assay.

**Fig. 3:**
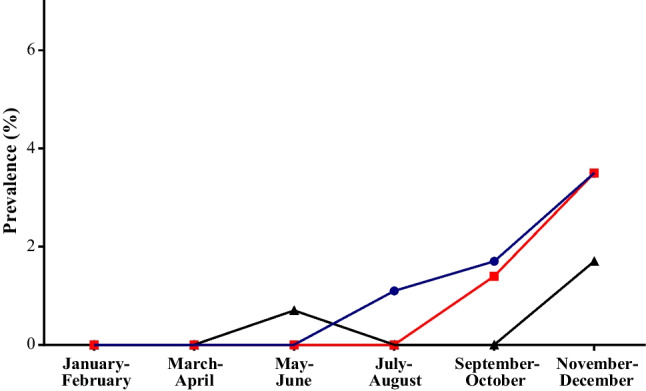
Prevalence by antibody class. Continued lines indicate RBD IgG (blue circle), RBD IgM (black triangle) and neutralizing antibody (red square)

When estimating the seroprevalence using a combination of the commercial ELISA and RBD-based ELISA/MN assay results, the total prevalence was 1.1% for RBD IgG, 0.5% for RBD IgM, and 0.9% for neutralizing antibody. The seroprevalence over the bimesters by the RBD antibody class and neutralizing antibody is shown in Fig. 3. The prevalence of both RBD IgG and neutralizing antibody increases from the second half of the year, reaching a value of 3.5% in the last bimester.

## Discussion

This study evaluates the SARS-CoV-2 antibody prevalence in a sample population in the province of Bari, in the Apulia region of Southern Italy, from January to the end of December 2020, before vaccine was deployed to the general population. Overall, 7.55% of the samples had IgM and/or IgG antibodies against SARS-CoV-2 when tested by commercial ELISA. While the proportion of IgM positive samples remained quite similar during the year, the proportion of samples with SARS-CoV-2 IgG antibodies increased from 1.9% in January–February to 9.6% in November–December. The findings of this study show that seroprevalence to SARS-CoV-2 in the province of Bari had an increasing trend during 2020, with a higher prevalence in the last months, in line with the epidemiological trend of the region. In fact, data obtained from the surveillance system show that as 4 May, the number of positive cases registered in the province of Bari from the start of the pandemic was 1328. When the Apulia region was labelled as an “orange zone” on 6 November, the number of positive cases in the province of Bari was 9669, reaching 35,127 cases by the end of the year (Presidenza del Consiglio dei Ministri 2020).

Despite the high number of cases registered in the second pandemic wave that occurred from September onwards, the prevalence values found in this study are relatively low, albeit consistent with those observed in other studies conducted in Southern Italy, which observed prevalence values between 4.7% and 5.8% (Napolitano et al. 2021; Cerino et al. 2021; Polvere et al. 2021). The low prevalence observed in the first months of 2020 can be explained by the extensive restrictive measures implemented on the population during the first pandemic wave. On the other hand, the relatively higher values in terms of prevalence and registered cases observed in the second wave may be explained by the fact that the Apulia region was labelled an “orange zone”, meaning that only a few containment measures were implemented. For example, greater levels of intra-regional mobility, school attendance and opening of some commercial activities were allowed than in the red zone, which was more similar to the lockdown imposed during the first wave (Presidenza del Consiglio dei Ministri - Governo Italiano 2020). Furthermore, data from countries that adopted strict lockdown measures indicate a low prevalence in the general population, comparable to those where very light restrictions were adopted (Fiore et al. 2021).

These results highlight that, despite the high number of cases, the majority of the population was still immunologically naïve to the virus, and thus it is not possible to consider that immunity following natural infection led to a sharp reduction in susceptible individuals and slowed the transmission of infection. This is even more evident when considering that only a small proportion (12%) of samples showing IgM and/or IgG antibodies by commercial ELISA had neutralizing antibodies. The increased prevalence of neutralizing antibody in the second half of the year, reaching the highest value (3.5%) in the last bimester of the year, is still clearly insufficient to provide herd immunity.

SARS-CoV-2 infection elicits an antibody response that predominantly targets the S protein and its RBD (Zhao et al. 2020). In this study, the immune response to SARS-CoV-2 was characterized by means of RBD ELISA and MN assay. Among samples with antibodies against the RBD, 61.1% also exhibited neutralizing antibodies, supporting the fact that antibodies directed against RBD of the S protein are highly neutralizing (Mazzini et al. 2021).

The use of residual samples is a limitation of this study, as it may not be completely representative of the population. Moreover, since not all SARS-CoV-2-infected individuals will develop antibodies or neutralizing antibody titers at detectable levels, our results may represent an underestimation of the proportion of individuals who have been infected with SARS-CoV-2 (den Hartog et al. 2020; Marchi et al. 2021; Milani et al. 2020). On the other hand, the determination of neutralizing antibody using a live SARS-CoV-2 strain may represent a key strength of this study, providing information on previous exposure to SARS-CoV-2 as well as on immunity to the virus.

To our knowledge, very few studies have considered the entire year of 2020, the pandemic year in the pre-vaccination era (Marchi et al. 2022; Eskild et al. 2022; Charlton et al. 2021). This study contributes to outlining the epidemiological and immunological situation of the SARS-CoV-2 pandemic in Italy in relation to the spread of the virus and the effects of the containment measures undertaken during the first year of the pandemic.

## Supplementary Information

ESM 1 (DOCX 122 kb)
