# Supplementary material for: SARS-CoV-2 epidemiological trend before vaccination era: a seroprevalence study in Apulia, Southern Italy, in 2020
Source: Z Gesundh Wiss. 2023 Feb 4:1–6. Online ahead of print. doi: 10.1007/s10389-023-01834-3 (PMC9898852; doi:10.1007/s10389-023-01834-3)
Supplement: Supplementary file 1 — (DOCX 122 kb) [file 10389_2023_1834_MOESM1_ESM.docx]

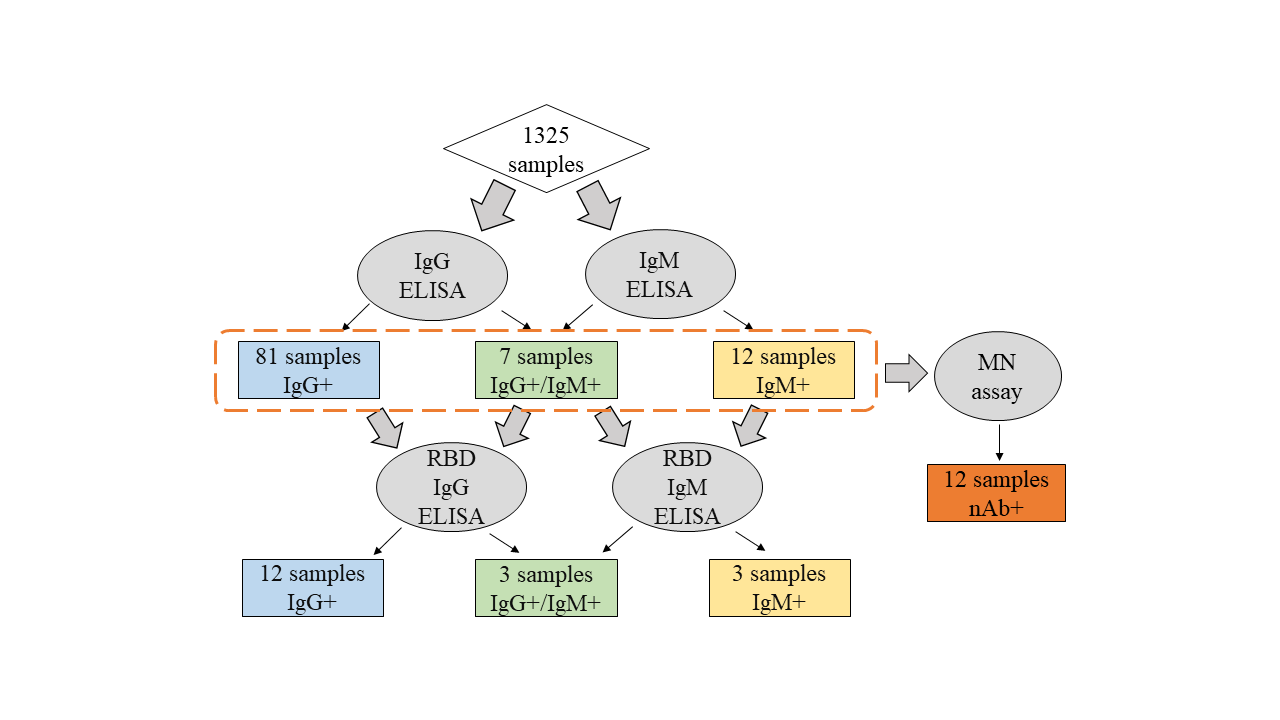


**Figure S1.** Flowchart describing the number of samples selected and tested by serological assay. Overall, 1325 samples were selected and tested by commercial ELISA for the detection of IgM and IgG antibodies against SARS-CoV-2. Positive samples were further tested by in-house ELISA for the detection of IgG and IgM against the receptor-binding domain (RBD) of the spike protein and by micro-neutralization (MN) assay for the detection of neutralizing antibody (nAb). Thick gray arrows indicate the serological assay by which the samples were tested, thin black arrows indicate the number of positive samples obtained with the test.
